# Lightweight Deep Learning Model for Classification of Normal and Abnormal Vasculature in Organoid Images

**DOI:** 10.3390/s26010112

**Published:** 2025-12-24

**Authors:** Eunsu Yun, Jongweon Kim, Daesik Jeong

**Affiliations:** 1 Department of Computer Science, Sangmyung University, Seoul 03016, Republic of Korea; hkhk0331@gmail.com; 2 Gyedang College of General Education, Sangmyung University, Seoul 03016, Republic of Korea

**Keywords:** human organoid, vasculature, classification model, EfficientNet, lightweight model, deep learning, organoid analysis, embedded system

## Abstract

Human organoids are 3D cell culture models that precisely replicate the microenvironment of real organs. In organoid-based experiments, assessing whether the internal vasculature has formed normally is essential for ensuring the reliability of experimental results. However, conventional vasculature assessment relies on manual inspection by researchers, which is time-consuming and prone to variability caused by subjective judgment. This study proposes a lightweight deep learning model for automatic classification of normal and abnormal vasculature in vascular organoid images. The proposed model is based on EfficientNet by replacing the activation function SiLU with ReLU and removing the Squeeze-and-Excitation (SE) blocks to reduce computational complexity. The dataset consisted of vascular organoid images obtained from co-culture experiments. Data augmentation and noise addition were performed to alleviate class imbalance. Experimental results show that the proposed Modified 3 models (B0, B1, B2) achieved accuracy of 0.90, 0.99, and 1.00, respectively, with corresponding inference speed of 51.1, 36.0, and 32.4 FPS on the CPU, demonstrating real-time inference capability and an average speed improvement of 70% compared to the original models. This study presents an efficient automated analysis framework that enables quantitative and reproducible vasculature assessment by introducing a lightweight model that maintains high accuracy and supports real-time processing.

## 1. Introduction

Human organoids are three-dimensional (3D) cell culture models that precisely replicate the microenvironment and physiological functions of human organs. They have become essential tools in various life science research fields, including drug efficacy and toxicity evaluation, disease modeling, and personalized medicine. Organoids can replicate cell–cell interactions, signal transduction, and tissue-specific morphogenesis, offering human-like physiological characteristics that are challenging to achieve with conventional two-dimensional (2D) cell cultures or animal models [1,2,3]. In particular, organoids containing vasculature play a crucial role in improving the reliability of preclinical research by accurately modeling complex physiological processes such as drug delivery, tumor growth, and immune responses. To ensure accurate drug response prediction and reliable experimental outcomes, it is essential to assess whether the internal vasculature of the organoid has formed normally. Abnormal vasculature may indicate issues in the experimental environment or the toxicity of specific drugs. Therefore, it serves as an important quality indicator that determines the success of organoid-based experiments [4,5,6].

Conventional assessment of vasculature mainly relies on visual inspection and manual image analysis performed by researchers [7,8]. Such manual assessments are time-consuming and require considerable labor, and they are fundamentally limited in scalability to high-throughput experimental settings due to variability caused by researchers’ skill and subjective judgment. Therefore, developing an automated system capable of objectively and reproducibly assessing normal and abnormal vasculature is an urgent requirement.

Recently, the application of deep learning techniques to organoid image analysis has gained increasing attention, showing significant potential. However, most prior studies focus on large-scale models with complex network architectures that demand high computational resources. For practical deployment in real-world laboratory environments or in systems with limited computational resources, such as embedded systems, computational efficiency and model lightweighting are key considerations.

In this study, we propose a lightweight deep learning model that efficiently analyzes vascular organoid images to automatically classify normal and abnormal vasculature. This model aims to address the limitations of previous approaches and maximize applicability in real experimental environments.

The main contributions of this study are as follows:

1. Proposal of an ultra-lightweight model architecture: Based on the high-performing EfficientNet architecture, a new lightweight model is designed by replacing the computationally expensive activation function SiLU with ReLU and removing the Squeeze-and-Excitation (SE) blocks, resulting in substantial reductions in parameters and computational complexity.
2. Solving data imbalance and generalization problems: For the imbalanced vascular organoid image dataset obtained from actual co-culture experiments, effective data augmentation and noise addition techniques are applied to enhance generalization performance and mitigate overfitting.
3. Validation of lightweighting effects and performance: The proposed model demonstrates comparable or even superior classification accuracy to the original model, as verified by quantitative metrics such as accuracy and F1-score. This study presents an automated analysis framework that enables quantitative and reproducible assessment of normal and abnormal vasculature.

The remainder of the paper is organized as follows. Section 2 reviews prior research on deep learning-based organoid image analysis and lightweight deep learning model. Section 3 describes the proposed model architecture, dataset construction, and training methods. Section 4 presents experimental results and performance comparisons. Section 5 concludes the paper.

## 2. Related Work

### 2.1. Organoid Image Analysis Using Deep Learning

Recently, deep learning-based approaches for organoid image analysis have been actively investigated. Wang et al. [9] proposed an organoid segmentation model using U-Net-based RDAU-Net to utilize bladder cancer-derived organoid images for high-throughput drug screening. Park et al. [10] developed OrgaExtractor, a multi-scale U-Net-based deep learning image processing tool, to precisely segment organoid morphologies of various sizes. Lefferts et al. [11] employed a Mask R-CNN-based segmentation model to recognize organoid objects and accurately delineate their boundaries. Wang et al. [12] proposed OrgSegNet, a U-Net-based segmentation model for analyzing the internal structure of human organoids using OCT images. Ong et al. [13] constructed an organoid segmentation and cellular topology analysis pipeline combining a 3D StarDist-based nuclei segmentation model with traditional image processing algorithms to rapidly analyze organoid structure. In addition, various studies have reported deep learning applications in organoid image analysis [14,15], and the scope of research continues to expand. These prior studies demonstrate the effectiveness of deep learning techniques in organoid image analysis. However, most prior studies are based on large-scale models with complex network architectures which require high computational resources. In environments that demand real-time analysis in real-world laboratory settings or operate under limited computational resources, such as embedded systems, the application of these large-scale models remains challenging.

### 2.2. Lightweight Deep Learning Model

#### 2.2.1. EfficientNet

EfficientNet is a convolutional neural network (CNN) proposed by Tan and Le in 2019, designed to balance accuracy and computational efficiency through a compound scaling strategy that simultaneously adjusts the depth, width, and resolution of the model [16]. While conventional CNN models typically improve performance by increasing depth or expanding width, EfficientNet achieves high accuracy with fewer parameters by optimally scaling these three elements together. The basic structure of EfficientNet is based on the Mobile Inverted Bottleneck Convolution (MBConv) block. The MBConv block is a structure first proposed by MobileNetV2 [17]. The MBConv block consists of an expansion stage that expands the channels of the input feature map, a depthwise convolution stage that performs convolution independently for each channel, and a projection stage that reduces the channel dimension again. EfficientNet incorporates a Squeeze-and-Excitation (SE) within each MBConv block to learn inter-channel dependencies. The SE block extracts channel-wise statistics through global average pooling (GAP) of the input feature map, computes channel-wise importance through two fully connected (FC) layers and an activation function. The calculated weights are multiplied by the input feature map to enhance the representation of important channels and suppress the contribution of unnecessary channels. Additionally, EfficientNet uses the SiLU (Sigmoid Linear Unit) activation function to provide smooth nonlinearity according to input values and mitigate gradient vanishing to enhance learning stability.

**Figure 1:**
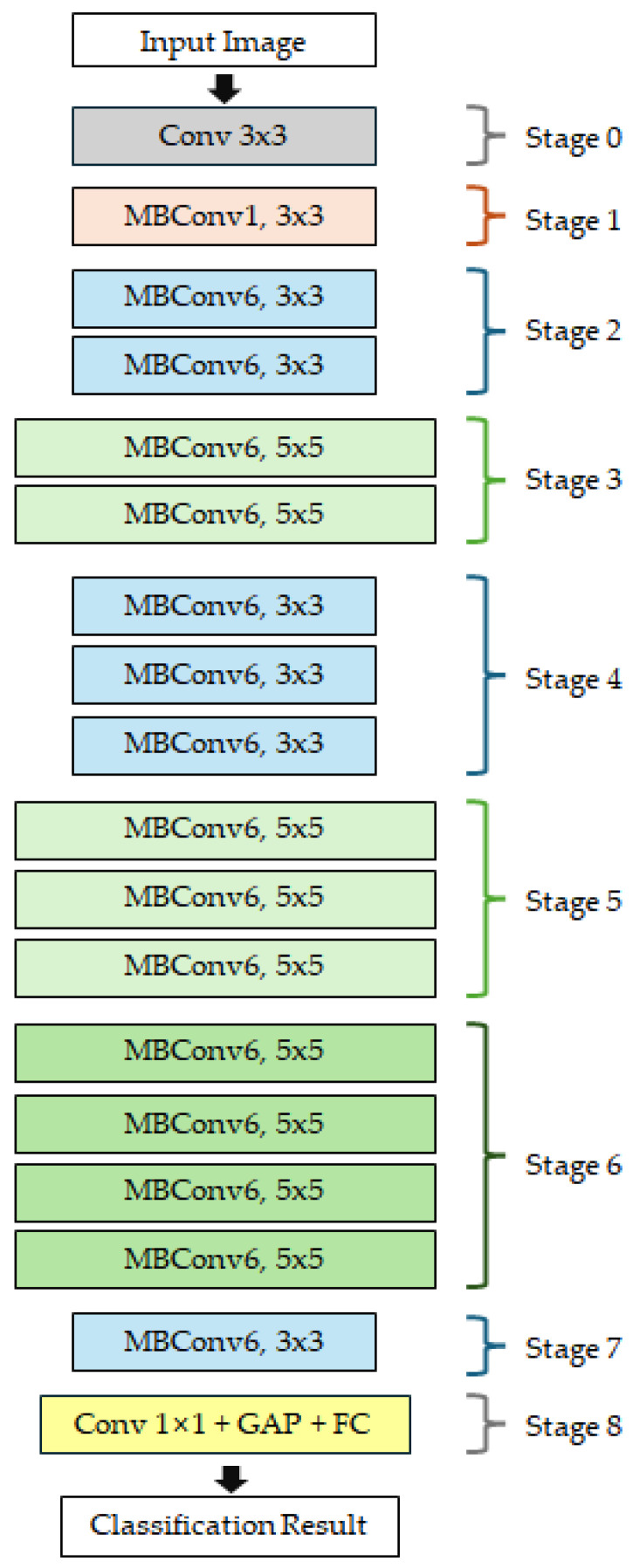
Overall architecture of the EfficientNet-B0 model, the baseline model of the EfficientNet series.

Figure 1 illustrates the overall architecture of EfficientNet-B0 model, the baseline model of the EfficientNet series. The input image passes through a network composed of a total of 9 stages (Stage 0–8). Stage 0 consists of a 3 × 3 convolution layer that extracts low-level features from the input image. Stages 1–7 consist of MBConv blocks with varying kernel sizes (3 × 3 or 5 × 5) and expansion ratios (1 or 6), enabling the model to progressively capture multi-scale features. Stage 8 comprises a 1 × 1 convolution layer, global average pooling (GAP), and a fully connected (FC) layer, which integrates the extracted high-level features and outputs the final classification result. The EfficientNet series expands from B0 to B7 by adjusting the compound scaling coefficient based on B0 as the basic architecture. In this study, EfficientNet-B0, B1, and B2 models were selected as baseline models considering the small-scale dataset and real-time inference environment.

#### 2.2.2. Squeeze-And-Excitation (SE) Block

**Figure 2:**
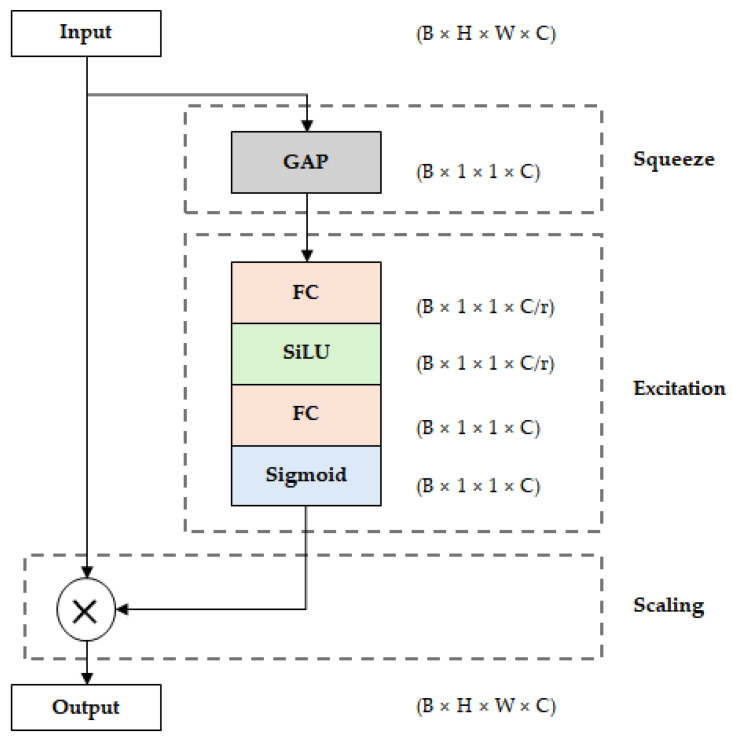
Structure of the Squeeze-and-Excitation (SE) block used in EfficientNet, illustrating the squeeze, excitation, and scaling stages for channel-wise feature recalibration.

The Squeeze-and-Excitation (SE) block [18] is a module that recalibrates feature maps by learning inter-channel dependencies. The structure of the SE block is shown in Figure 2. First, in the squeeze stage, global average pooling (GAP) is performed on the input feature map to extract channel-wise statistics. Then, in the excitation stage, channel-wise weights are calculated through two fully connected (FC) layers and an activation function. In the first FC layer, the channel reduction ratio (r) is applied to reduce the number of parameters, and in the second FC layer, it is expanded back to the original number of channels. At this time, SiLU is used as the activation function of the first FC layer, and the sigmoid function is used in the second layer to normalize the weight values between 0 and 1. Finally, in the scaling stage, these calculated channel-wise weights are multiplied by the original feature map to enhance the representation of important channels and suppress the contribution of unnecessary channels. In EfficientNet, the SE block is incorporated within each MBConv block and operates in combination with convolution operations. While the SE block improves feature representation, it also increases the number of parameters and computational cost due to the additional FC layers and operations.

#### 2.2.3. Activation Function

In deep learning models, the activation function is a core component that determines the nonlinearity of the network, directly affecting the model’s feature representation capability, learning stability, performance, and computational efficiency. This section compares the characteristics of SiLU used in EfficientNet and ReLU widely used in lightweight models.

SiLU (Sigmoid Linear Unit) [19] is defined as the product of the input value x and the Sigmoid function $\sigma \left(x\right)$ and is expressed as Equation (1).

(1) $$f\left(x\right)=x\cdot \sigma \left(x\right)=\frac{x}{1+e^{-x}}$$

SiLU provides continuous and smooth output according to input values and mitigates the vanishing gradient problem by maintaining small gradients even for negative inputs. Due to these characteristics, SiLU is advantageous for learning stability and performance improvement. However, since it includes sigmoid operations, additional exponential computations are required, which reduces computational efficiency in environments with limited hardware resources.

ReLU (Rectified Linear Unit) [20] is a simple conditional-based nonlinear function that outputs x when the input value x is greater than 0 and outputs 0 otherwise. ReLU is expressed as Equation (2).

(2) $$f\left(x\right)=max\left(0,x\right)$$

ReLU is implemented with simple operations and has very low computational complexity, significantly improving inference speed. Therefore, it is widely used in lightweight models and real-time processing environments where the computational efficiency of hardware is critical.

## 3. Materials and Methods

### 3.1. Proposed Lightweight Model

EfficientNet is a model that achieves high accuracy with fewer parameters and lower computational cost compared to existing CNN models on large-scale image classification datasets such as ImageNet. However, this study aims to enable real-time analysis in actual experimental environments, which requires additional model lightweighting. Particularly in environments with limited computational resources, such as embedded systems, the model’s computational efficiency becomes a key consideration. In such environments, the complex computational structure of the Squeeze-and-Excitation (SE) block and the high computational cost of the SiLU activation function may reduce computational efficiency. Therefore, in this study, we set EfficientNet-B0, B1, and B2 models as baseline models and designed a lightweight architecture with reduced computational complexity by removing SE blocks and replacing the activation function.

**Figure 3:**
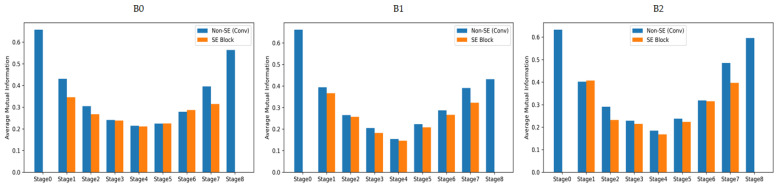
Comparison of mutual information (MI) between sections with SE blocks and without SE blocks across different stages of EfficientNet-B0, B1, and B2 models.

The Squeeze-and-Excitation (SE) block in EfficientNet includes two fully connected (FC) layers and sigmoid operations, which increase the number of parameters and computational cost. These structural characteristics can degrade inference speed in environments with limited computational resources, such as embedded systems. In this study, we conducted a mutual information (MI) analysis to quantitatively evaluate the amount of useful information provided by the SE block in this dataset. MI is an information-theoretic metric that measures the interdependence between two random variables, indicating the amount of shared information between them. A higher value represents stronger dependency between input features and outputs, while a lower value indicates limited information contribution [21]. We compared MI values between sections with and without SE blocks in each stage of EfficientNet-B0, B1, and B2 models to measure how much mutual information they share with the output labels. The Scikit-learn library [22] was used to calculate the MI values, and the results for each model are shown in Figure 3. Analysis results showed that in all EfficientNet-B0, B1, and B2 models, the MI values of sections with SE blocks were lower or nearly identical to those without SE blocks across most stages. This indicates that SE blocks do not significantly contribute to improving discriminative power in terms of mutual information with output labels. Accordingly, this study designed a lightweight architecture that enhances computational efficiency by removing SE blocks.

**Figure 4:**
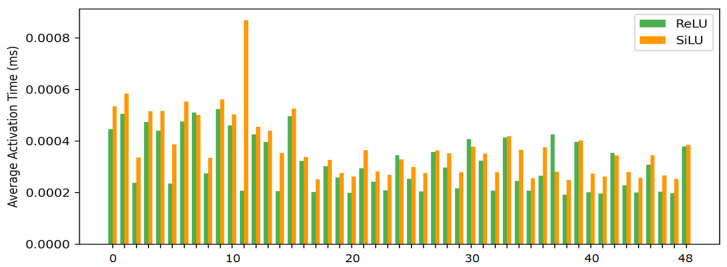
Comparison of computational speed between ReLU and SiLU for each of the 49 activation operations in EfficientNet-B0 on the CPU.

EfficientNet uses the SiLU (Sigmoid Linear Unit) activation function. However, SiLU with Sigmoid operation has high computational cost due to exponential operation. In this study, we observed that EfficientNet-B0 model performs a total of 49 activation operations. Figure 4 presents the measured computational speeds of the activation functions in a CPU environment. The results indicate that SiLU is, on average, approximately 1.25 times slower than ReLU in processing speed. Accordingly, this study designed a lightweight architecture that improves computational efficiency by replacing SiLU with ReLU, making it applicable to embedded systems requiring real-time inference.

In this study, we designed lightweight models based on EfficientNet-B0, B1, and B2 models. The proposed model aimed to reduce computational complexity while achieving similar or improved classification performance through activation function replacement and Squeeze-and-Excitation (SE) block removal. Three model variants were constructed according to the modification method, and their configuration and parameter comparisons are summarized in Table 1. The characteristics of each model are as follows.

- Modified 1: A variant that replaces the original EfficientNet’s activation function SiLU with ReLU. Since only the activation function replacement was applied, there is no change in the total number of parameters and model size.
- Modified 2: A variant that removes the SE blocks within the original EfficientNet’s MBConv blocks. By removing them, the number of parameters and model size decreased overall. Based on EfficientNet-B0 model, the number of parameters decreased by about 15% from 4.0 M to 3.4 M, and the model size decreased by about 15% from 16.3 MB to 13.8 MB.
- Modified 3: A variant that applies both activation function replacement and SE block removal simultaneously. The number of parameters and model size are the same as Modified 2. The experimental results showed that Modified 3 was the most efficient architecture among the three modified models.

**Figure 5:**
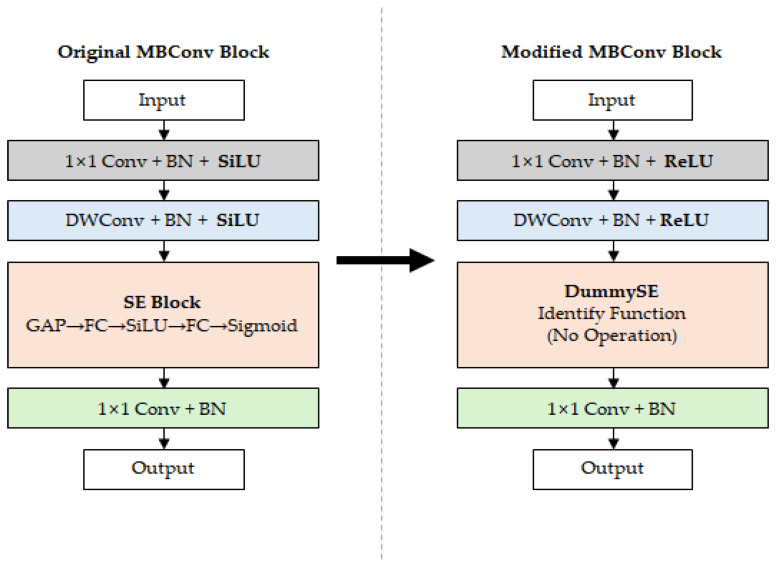
Comparison of the MBConv block structures of the original EfficientNet and the proposed Modified 3 model.

Figure 5 shows the MBConv block structure of the original EfficientNet and the proposed Modified 3 model. The Original MBConv block on the left includes the SiLU activation function and SE block, with global average pooling (GAP), fully connected (FC) layers, SiLU, and Sigmoid operations inside the SE block. In contrast, the Modified MBConv block on the right replaced SiLU with ReLU and removed the SE block to reduce computational complexity. Additionally, SE blocks were replaced with dummy functions (DummySE) to maintain the computational flow of the network architecture.

### 3.2. Dataset and Preprocessing

**Figure 6:**
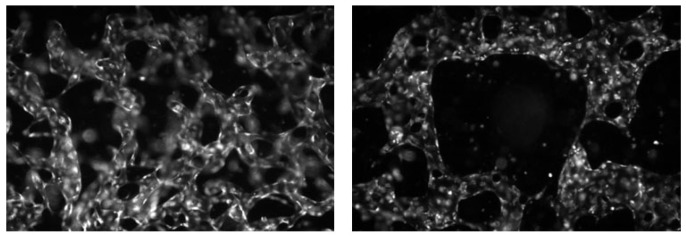
Representative examples of normal and abnormal vasculature in organoid images.

The data used in this study were provided by the Korea Basic Science Institute (KBSI), where both image acquisition and labeling were performed by domain experts. The normal and abnormal labels were assigned based on vascular morphological characteristics. In these experiments, breast cancer spheroids, human umbilical vein endothelial cells (HUVEC), and breast fibroblasts were co-cultured within the same microfluidic chip to replicate the tumor microenvironment. Fixed samples were imaged using a fluorescence microscope, and the acquired data were saved as TIFF-format multichannel images. In this study, only the channel representing vasculature was separated and used among all fluorescence channels. Representative examples of normal and abnormal vasculature are shown in Figure 6.

The original dataset consists of a total of 1142 images. The dataset was divided into training, verification, and test sets. As shown in Table 2, the entire dataset consists of 169 normal and 973 abnormal images. Among these, the training set was divided into 80 normal and 884 abnormal images, the validation set into 21 normal and 21 abnormal images, and the test set into 68 normal and 68 abnormal images.

To solve the class imbalance problem, data augmentation was applied only to the normal class in the training set, and no augmentation was applied to the validation and test sets. Specifically, geometric transformation techniques such as horizontal and vertical flipping and rotation at various angles were applied. Rotation angles were set at 15°, 30°, 60°, 90°, 180°, and 270°. Additionally, image zooming was applied for scale transformation, with magnification ratios set at +5% and +10%. This is a typical approach to improve learning stability and generalization performance by applying various geometric transformations to classes with limited data [23,24].

In addition, Gaussian noise was applied to the normal and abnormal classes of the training set to generate additional images. Specifically, Gaussian noise with zero mean was used, and the standard deviation was randomly sampled between 0.02 and 0.06 and added to each image. This approach helps the model avoid overfitting to specific patterns and achieve more robust generalization performance against various variabilities that may occur in real-world imaging conditions [25].

All images underwent a series of preprocessing steps before training. Original images were resized to 224 × 224 pixels to match the model’s input size, and single-channel images were converted to three channels (RGB). Additionally, all pixel values were normalized to the [−1, 1] range. These preprocessing settings were selected based on the characteristics of lightweight models. The 224 × 224 resolution, which is the standard input size for EfficientNet, provides a balance between computational efficiency and sufficient feature representation for real-time processing. Normalizing pixel values to the [−1, 1] range centers the data distribution around zero and improves training stability, which is particularly beneficial for lightweight models with fewer parameters.

### 3.3. Evaluation Metrics

In this study, four main classification metrics were used to quantitatively evaluate the performance of the proposed model: accuracy, precision, recall, and F1-score [26].

Accuracy represents the proportion of correctly classified samples among all samples and is the most intuitive metric showing the overall performance of a classification model. The definition of accuracy is given in Equation (3).

(3) $$Accuracy=\frac{\left(TP+TN\right)}{\left(TP+TN+FP+FN\right)}$$

Precision represents the proportion of actual positives among samples classified as positive, evaluating how many false positives (FP) predictions were reduced. The definition of precision is given in Equation (4).

(4) $$Precision=\frac{TP}{\left(TP+FP\right)}$$

Recall represents the proportion of correctly classified samples among actual positive samples, evaluating how many false negatives (FN) predictions were reduced. Recall is also called sensitivity, and its definition is given in Equation (5).

(5) $$Recall(Sensitivity)=\frac{TP}{\left(TP+FN\right)}$$

F1-score is the harmonic mean of precision and recall, a metric evaluating the balance between the two metrics. It is particularly useful in imbalanced dataset environments and becomes an important performance metric when there is a difference in the amount of data between normal and abnormal as in this study. The definition of F1-score is given in Equation (6).

(6) $$F1-score=\frac{2\;*\;(Precision\;*\;Recall)}{\left(Precision+Recall\right)}$$

In addition, t-distributed Stochastic Neighbor Embedding (t-SNE) [27] was used to visually analyze the high-dimensional feature representations generated by the model during training. t-SNE is a dimensionality reduction technique that projects high-dimensional feature representations into 2D or 3D, allowing visual evaluation of class separation. This allows us to confirm how effectively normal and abnormal vasculature are separated in the representation space.

### 3.4. Experimental Setup

All experiments and model training in this study were conducted in an Ubuntu 20.04 LTS operating system and Python 3.8 environment. The hardware configuration consists of an AMD Ryzen 9 5900X (12-Core) CPU (Advanced Micro Devices, Santa Clara, CA, USA) and NVIDIA GeForce RTX 3090 GPU (NVIDIA Corporation, Santa Clara, CA, USA). The software environment was configured based on PyTorch 2.2.2, Torchvision 0.17.2, and CUDA 12.4. The hardware and software environment configuration used in the experiments is organized in Table 3.

Model training was conducted for a total of 50 epochs, with the initial learning rate set at 1 × 10^−4^. AdamW was used as the optimization algorithm, and cosine annealing was applied as the learning rate scheduler. The batch size was set at 32, and the dropout ratio was set at 0.2.

## 4. Results and Discussion

### 4.1. Convergence Analysis

**Figure 7:**
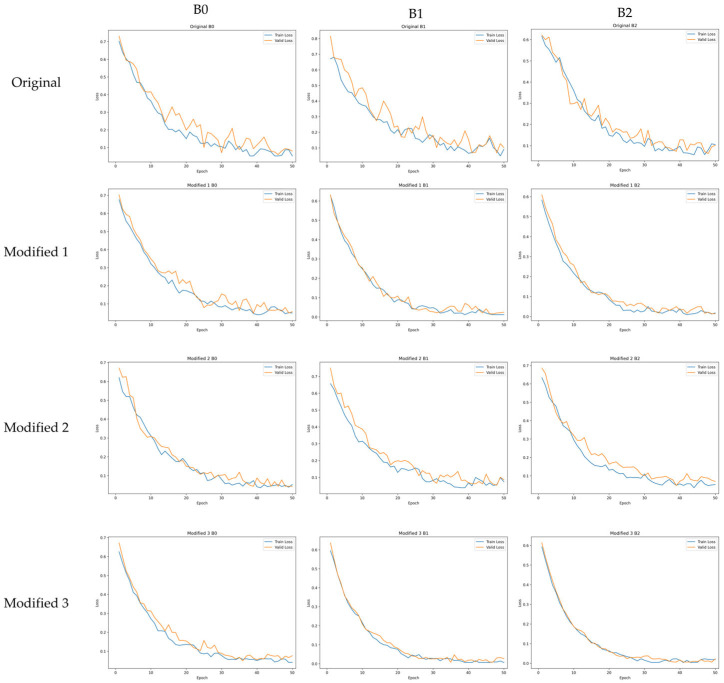
Training and validation loss curves of the original models and the proposed Modified models.

Figure 7 presents the training and validation loss curves of the original models and the proposed Modified models. The original models show large fluctuations in validation loss throughout the training process, and the loss reduction progresses relatively slowly. In contrast, the proposed Modified 3 model exhibits a rapid decrease in loss during the early training stage and maintains a stable convergence pattern with minimal oscillation thereafter. This behavior can be attributed to the lightweighting strategy applied in this study, which simplifies the model structure, reduces optimization complexity, and enables more consistent loss reduction. The Modified 1 and Modified 2 models also show reduced fluctuations in validation loss compared with the original models due to the activation function replacement and SE block removal, although the degree of improvement in training stability is less pronounced than that of the Modified 3 model. Overall, the Modified 3 model demonstrates faster convergence speed and enhanced training stability compared with the original models, which aligns with the improved classification performance presented in Section 4.2. These results indicate that the proposed model is more advantageous than the original models in terms of training efficiency and practical applicability.

### 4.2. Classification Performance

**Figure 8:**
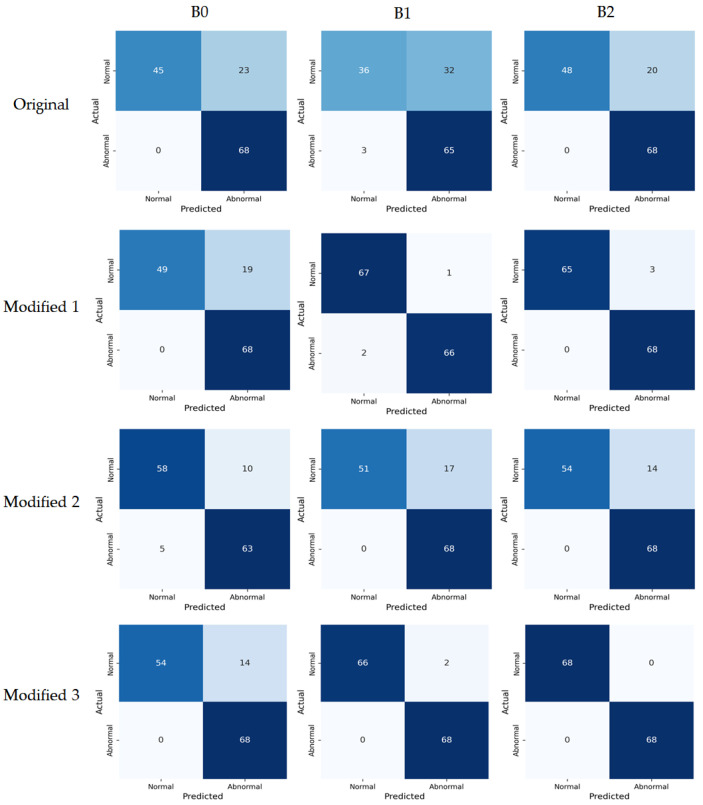
Confusion matrices of the original models and the proposed Modified models on the test set. The color intensity represents the number of samples, with darker blue indicating higher values.

Model performance metrics were calculated based on the confusion matrix using the test set. Figure 8 presents the confusion matrices visualizing each model’s prediction results, showing the classification outcomes between the actual and predicted labels. Model classification performance was compared based on the accuracy metric, and the results are summarized in Table 4. Overall, the proposed Modified models consistently outperformed the original models. The Modified 1 model, which only replaced the activation function, achieved accuracy of 0.86, 0.98, and 0.98 for B0, B1, and B2, respectively, demonstrating overall superior performance compared to the original models. The Modified 2 model simplified the architecture by removing SE blocks and achieved accuracy of 0.89, 0.88, and 0.90 for B0, B1, and B2, respectively, maintaining higher performance than the original models. Finally, the Modified 3 model, which applied both activation function replacement and SE block removal, achieved accuracy of 0.90, 0.99, and 1.00 for B0, B1, and B2, respectively, showing the best performance among all models. These results indicate that activation function replacement and SE block removal not only reduced computation but also contributed to improving generalization performance by preventing unnecessary feature learning.

While accuracy is a representative metric for assessing overall classification performance, it alone is insufficient to fully explain model performance when class imbalance exists in the dataset. Therefore, in this study, we comprehensively analyzed balanced performance between classes by evaluating precision, recall, and F1-score together. Class-wise precision, recall, and F1-score are presented in Table 5. Overall, the proposed Modified models showed more stable and balanced performance than the original models. For the original models, recall was low for the Normal class, showing a tendency to misclassify normal samples as abnormal (false negatives), and precision was low for the Abnormal class, meaning many samples predicted as abnormal were actually normal (false positives). The proposed Modified models improved precision and recall for both normal and abnormal classes, mitigating the class imbalance problem, and secured balanced classification performance in F1-score as well. However, the recall for the normal class was still relatively lower than the abnormal class, indicating that some tendency to misclassify normal samples as abnormal remained.

**Figure 9:**
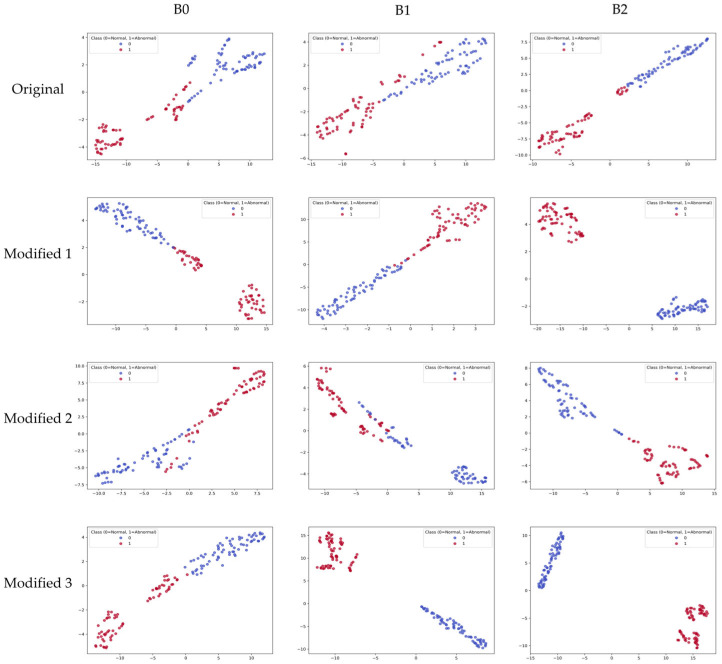
t-SNE visualization of feature embeddings extracted from the original models and the proposed Modified models on the test set.

To visually analyze the feature representation distribution of the model, the results of projecting learned embeddings into 2D space using t-distributed Stochastic Neighbor Embedding (t-SNE) are shown in Figure 9. The t-SNE visualization was computed using the feature embeddings extracted from the layer immediately before the final FC layer, using all samples in the test set. In the original models, some overlap between normal and abnormal classes was observed, and class boundaries were not clear. In the proposed Modified models, Modified 1 and Modified 2 still showed partial overlap between normal and abnormal classes. Nevertheless, they appeared more clearly separated compared to the original models. In contrast, the Modified 3 model appeared in clearly separated cluster forms, showing the most distinct separation among all variants. These results mean that the proposed lightweighting strategy contributed not only to improving computational efficiency but also to improving discriminative power in the feature space.

### 4.3. Computational Efficiency

The computational efficiency of the model was evaluated based on inference speed (Latency, FPS) on the CPU and GPU environments, and the results are summarized in Table 6. Latency is the average inference time for a single image, and FPS (Frames Per Second) is the number of images processed per second. These two metrics are key indicators for determining the practical applicability of lightweight models and are particularly important in environments with limited computational resources, such as embedded systems.

Overall, the proposed Modified models showed superior inference speed compared to the original models. This shows that the lightweighting strategy through activation function replacement and SE block removal not only reduced the number of parameters but also led to actual computational efficiency improvement. The Modified 1 model reduced latency on the CPU by about 38% compared to the original models by replacing the activation function SiLU with ReLU, which is interpreted as a result of reduced computation by removing Sigmoid operations in the SiLU function. On the GPU, the speed improvement was relatively minimal because SiLU operations are processed in parallel. The Modified 2 model reduced latency on the GPU by about 40% compared to the original models by removing the SE block, and computational efficiency improved with latency reduced by about 10% compared to the original models even on the CPU. The Modified 3 model is a structure that simultaneously applied both methods, with latency reduced by about 40% on the CPU and about 38% on the GPU. In addition, the Modified 3 model recorded 51.1, 36.0, and 32.4 FPS for B0, B1, and B2, respectively, representing improvements of 74%, 71%, and 67% compared to the original models and demonstrating real-time inference capability. These results experimentally confirm that the proposed lightweighting strategy can significantly improve inference efficiency without compromising classification accuracy.

## 5. Conclusions

In this study, we proposed a lightweight deep learning model for automatic classification of normal and abnormal vasculature in vascular organoid images. Based on EfficientNet, we maintained high classification accuracy while reducing computational complexity by replacing the activation function SiLU with ReLU and removing the Squeeze-and-Excitation (SE) blocks. Using a vascular organoid image dataset obtained from co-culture experiments, the Modified 3 model achieved accuracy of 0.90, 0.99, and 1.00 for B0, B1, and B2, respectively. Furthermore, on the CPU environment, the Modified 3 model recorded 51.1, 36.0, and 32.4 FPS for B0, B1, and B2, respectively, demonstrating real-time inference capability and an average speed improvement of 70% compared to the original models. These results experimentally confirm that the proposed lightweighting strategy not only reduces the number of parameters but also contributes to both improved inference efficiency and enhanced discriminative performance.

The main significance of this study lies in presenting an efficient automated analysis framework that enables quantitative and reproducible assessment of normal and abnormal vasculature. The conventional vasculature assessment relies on researchers’ subjective judgment and has limitations in requiring considerable analysis time and human resources. In contrast, the proposed model can rapidly perform vasculature classification with minimal human intervention, making it applicable to high-throughput experimental environments and improving the reliability and efficiency of organoid-based experiments.

Although the proposed model demonstrated strong performance, two aspects require further verification to strengthen its practical applicability. First, the dataset used in this study was limited to specific experimental conditions and cell combinations, necessitating additional evaluation to assess generalization capability across different organoid types, imaging sessions, and acquisition conditions. Second, the model was evaluated only on CPU and GPU environments, and its performance on an actual embedded system has not yet been verified. Therefore, future work will expand the dataset to include diverse imaging conditions and biological variations, thereby improving the robustness and generalization capability of the model. In addition, we will further develop lightweighting through model quantization and inference optimization and evaluate the model on embedded systems. Through these efforts, the proposed model is expected to be extended for real-time analysis in real-world laboratory environments.

## Figures and Tables

**Table 1:** Comparison of model configurations and parameters of the original models and proposed Modified models.

Model Variant	Base	Activation Function	SE Block	Params (M)	Model Size (MB)
Original	B0	SiLU	✓	4.0	16.3
	B1			6.5	26.5
	B2			7.7	30.7
Modified 1	B0	ReLU	✓	4.0	16.3
	B1			6.5	26.5
	B2			7.7	30.7
Modified 2	B0	SiLU	✗	3.4	13.8
	B1			5.4	21.9
	B2			6.4	25.9
Modified 3	B0	ReLU	✗	3.4	13.8
	B1			5.4	21.9
	B2			6.4	25.9

✓ indicates the presence of the SE block; ✗ indicates the absence of the SE block.

**Table 2:** Summary of dataset construction and class distribution at each preprocessing stage.

Stage	Normal	Abnormal	Total
Original	169	973	1142
After augmentation	969	973	1942
After noise addition	1241	1142	2383

**Table 3:** Hardware and software environment specifications used for model training and evaluation.

Component	Specification
CPU	AMD Ryzen 9 5900X (12-Core) (Advanced Micro Devices, Santa Clara, CA, USA)
GPU	NVIDIA GeForce RTX 3090 (NVIDIA Corporation, Santa Clara, CA, USA)
OS	Ubuntu 20.04 LTS
Python	3.8
CUDA	12.4
Framework	PyTorch 2.2.2, Torchvision 0.17.2

**Table 4:** Classification accuracy of the original models and proposed Modified models on the test set.

Model Variant	Base	Accuracy
Original	B0	0.83
	B1	0.74
	B2	0.85
Modified 1	B0	0.86
	B1	0.98
	B2	0.98
Modified 2	B0	0.89
	B1	0.88
	B2	0.90
Modified 3	B0	0.90
	B1	0.99
	B2	1.00

**Table 5:** Class-wise precision, recall, and F1-score of the original models and the proposed Modified models on the test set.

Model Variant	Base	Normal			Abnormal		
		Precision	Recall	F1-Score	Precision	Recall	F1-Score
Original	B0	1.00	0.66	0.80	0.75	1.00	0.86
	B1	0.92	0.53	0.67	0.67	0.96	0.79
	B2	1.00	0.71	0.83	0.77	0.96	0.87
Modified 1	B0	1.00	0.72	0.84	0.78	1.00	0.88
	B1	0.97	0.99	0.98	0.99	0.97	0.98
	B2	1.00	0.96	0.98	0.96	1.00	0.98
Modified 2	B0	0.92	0.85	0.89	0.86	0.93	0.89
	B1	1.00	0.75	0.86	0.80	1.00	0.89
	B2	1.00	0.79	0.89	0.83	1.00	0.91
Modified 3	B0	1.00	0.79	0.89	0.83	1.00	0.91
	B1	1.00	0.97	0.99	0.97	1.00	0.99
	B2	1.00	1.00	1.00	1.00	1.00	1.00

**Table 6:** Inference speed (latency and FPS) of the original models and the proposed Modified models on the CPU and GPU environments.

Model Variant	Base	CPU		GPU	
		Latency (ms)	FPS	Latency (ms)	FPS
Original	B0	34.1	29.3	7.3	138.0
	B1	47.7	21.0	10.3	97.6
	B2	51.7	19.4	10.5	95.1
Modified 1	B0	21.1	47.4	7.1	140.0
	B1	29.5	34.0	10.3	97.8
	B2	32.0	31.3	10.5	95.3
Modified 2	B0	30.6	32.7	4.4	227.5
	B1	43.5	23.0	6.1	163.8
	B2	47.4	21.1	6.2	162.0
Modified 3	B0	19.6	51.1	4.5	224.3
	B1	27.8	36.0	6.3	158.4
	B2	30.9	32.4	6.4	155.6

## Data Availability

The data presented in this study are available on request from the corresponding authors. The data are not publicly available due to institutional data-sharing restrictions.
